# Analysis of the Complete Chloroplast Genome of a Medicinal Plant, *Dianthus superbus* var. *longicalyncinus*, from a Comparative Genomics Perspective

**DOI:** 10.1371/journal.pone.0141329

**Published:** 2015-10-29

**Authors:** Gurusamy Raman, SeonJoo Park

**Affiliations:** Department of Life Sciences, Yeungnam University, Gyeongsan, Gyeongsan-buk, Republic of Korea; National Renewable Energy Lab, UNITED STATES

## Abstract

*Dianthus superbus* var. *longicalycinus* is an economically important traditional Chinese medicinal plant that is also used for ornamental purposes. In this study, *D*. *superbus* was compared to its closely related family of Caryophyllaceae chloroplast (cp) genomes such as *Lychnis chalcedonica* and *Spinacia oleracea*. *D*. *superbus* had the longest large single copy (LSC) region (82,805 bp), with some variations in the inverted repeat region A (IRA)/LSC regions. The IRs underwent both expansion and constriction during evolution of the Caryophyllaceae family; however, intense variations were not identified. The pseudogene ribosomal protein subunit S19 (*rps19*) was identified at the IRA/LSC junction, but was not present in the cp genome of other Caryophyllaceae family members. The translation initiation factor IF-1 (*infA*) and ribosomal protein subunit L23 (*rpl23*) genes were absent from the *Dianthus* cp genome. When the cp genome of *Dianthus* was compared with 31 other angiosperm lineages, the *infA* gene was found to have been lost in most members of rosids, solanales of asterids and *Lychnis* of Caryophyllales, whereas *rpl23* gene loss or pseudogization had occurred exclusively in Caryophyllales. Nevertheless, the cp genome of *Dianthus* and *Spinacia* has two introns in the proteolytic subunit of ATP-dependent protease (*clpP*) gene, but *Lychnis* has lost introns from the *clpP* gene. Furthermore, phylogenetic analysis of individual protein-coding genes *infA* and *rpl23* revealed that gene loss or pseudogenization occurred independently in the cp genome of *Dianthus*. Molecular phylogenetic analysis also demonstrated a sister relationship between *Dianthus* and *Lychnis* based on 78 protein-coding sequences. The results presented herein will contribute to studies of the evolution, molecular biology and genetic engineering of the medicinal and ornamental plant, *D*. *superbus* var. *longicalycinus*.

## Introduction

Chloroplasts are double membrane bound plant organelles that encode genes essential for photosynthesis and other biochemical pathways such as biosynthesis of starch, fatty acids, pigments and amino acids [1]. This organelle possesses its own single circular DNA, chromosome, which is highly conserved among species. Most chloroplasts carry two copies of inverted repeats (IRs) separated by a large single copy region (LSC) and a small single copy region (SSC). To date, more than 340 chloroplast (cp) genomes have been completely sequenced and characterized and are available in the Chloroplast Genome Database (http://chloroplast.ocean.washington.edu/tools/cpbase/run). The majority of angiosperm cp genome sequences are highly conserved, and these usually encode four rRNAs, 30 tRNAs and approximately 80 unique proteins. Previous studies reported that gene content, gene order and genome organization are highly conserved within terrestrial plants based on restriction site mapping [2,3]. However, with the availability of more chloroplast genomes in the database, comparative genome studies have been carried out. These investigations have revealed many structural gene rearrangements, large IR expression and the occurrence of gene loss in numerous angiosperm lineages [4,5]. Such studies are essential to reconstruction of plant phylogenetic trees [6], DNA barcoding [7], and population [8] and transplastomic studies [9].

Angiosperms are considered the most ancient flowering plant, originating approximately 160 million years ago [10]. The angiosperms consist of four major groups, basal angiosperms, magnoliids, monocots and eudicots. Caryophyllaceae is considered to be the most diverse and largest family of eudicots, consisting of 86 genera and 2,200 species [11–13]. These flowering groups of plants are widely distributed in the Mediterranean and bordering regions of Europe and Asia. The *Dianthus* genus consists of nearly 300 species native to Europe and Asia, with a few species extending to North Africa and arctic North America. The blooms of *D*. *superbus* are five petaled with green eyes. The petals are deeply notched, giving them a feathery or fringed appearance, leading to their commonly being referred to as fringed pink or large pink. *D*. *superbus* contains two species varieties, *longicalycinus* and *speciosus*. *D*. *superbus* var. *longicalycinus* is a herbaceous evergreen perennial plant that reaches 6–12 inches in height and is commonly grown in East Asian countries, especially China, Japan and Korea. *D*. *superbus* var. *longicalycinus* is a popular garden plant that has been used for its scent and as a Chinese herbal medicine (Qu Mai) for over 2,000 years. Specifically, it is commonly used as an anti-inflammatory agent for urinary infections, carbuncles and carcinoma of the esophagus [14,15]. The ethanol extract of *D*. *superbus* has been shown to suppress the production of IgE in a human B cell line, a murine model of peanut allergy, interleukin-4 (IL-4), IL-13 and eotaxin [16]. This medicinal herb stimulates the digestive and urinary systems, lowers blood pressure and reduces fever [16,17]. This plant also acts as an antibacterial agent, abortifacient, contraceptive, diuretic, emmenagogue, ophthalmic, tonic, and hair growth promoter and has the potential for use as an antifertility agent [17]. The plant is taken internally to treat acute urinary tract infections (especially cystitis), urinary stones, constipation and failure to menstruate [14]. It is applied externally to treat skin inflammation and swelling. The leaves are used in the treatment of hemorrhoids, lumbricoid worms, and venereal sores, while the flowers are used as an astringent, diuretic, hemostatic, resolvent and vulnerary [15].

Many genes have been lost from the chloroplast genome during plant evolution [18]. Martin *et al*. [19] reported that most of these losses happened in the interval between the original endosymbiosis of a cyanobacterium (containing ~2000 protein-coding genes) and the last common ancestor of all existing chloroplast genomes (~ 210 protein coding genes). Gene loss or pseudogenes were observed in several land plants [18]. The cell viability pseudogene, *ycf2*, in rice and maize [20,21], the ribosomal protein subunit L23 (*rpl23*) in spinach [22] and the translation initiation factor (*infA*) were observed in tobacco, *Arabidopsis* and *Oenothera elata* [23–26]. Previous studies showed that the chloroplast genes ribosomal protein subunit L22 (*rpl22*), ribosomal protein subunit s16 (*rps16*) and subunit of photosystem I gene, *ycf4*, have been lost from some or all legume plants of angiosperms [27,28]. Additionally, nicotinamide adenine dinucleotide (NADH) dehydrogenase F (*ndhF*) and *ycf2* were lost repeatedly from a variety of angiosperms [29–31]. Intron loss has occurred in the *clpP* (proteolytic subunit of ATP-dependent protease) gene of *Sileneae* [32]. Due to gene loss, pseudogenes, intron loss, inversions, shifts in inverted repeat boundaries and large insertions and deletions in the cp genome of land plants provide the most information about the evolutionary mechanisms involved.

Owing to lack of chloroplast genome information regarding this important medicinal and ornamental plant, there is demand to develop its genetic resources further. We previously sequenced and reported the cp genome of *Dianthus superbus* var. *longicalycinus* [33]. However, in this study, we characterized and analyzed the cp genome of *Dianthus* and conducted comparative genomics of its closely related family of Caryophyllaceae cp genomes such as *Lychnis chalcedonica* and *Spinacia oleracea*. The cp pseudogenes, *infA* and *rpl23*, and the intron containing *clpP* gene of *Dianthus* were analyzed and compared with 31 other angiosperm lineages to understand the evolutionary perspective of these genes. In addition, molecular phylogenetic analyses were conducted based on 78 protein-coding genes from 32 taxa. The results presented herein will contribute to a better understanding of the molecular biology, genetics and evolution of the *Dianthus* genus. In addition, these data should be useful for future studies of chloroplast genomes and phylogenomic studies of Caryophyllales.

## Materials and Methods

### Comparative genome analysis of the *Dianthus* chloroplast genome

The complete chloroplast genome of *D*. *superbus* var. *longicalycinus* was compared with that of three other species, *L*. *chalcedonica*, *S*. *oleracea* and *N*. *tabacum*. To visualize the genomes of the four cp species, the annotated cp genomes were aligned using the Mauve program [34] and plotted with Circos 0.67 [35] to show gene locations, GC skew and GC content. Moreover, the four cp genomes were compared with the mVISTA program in Shuffle-LAGAN mode [36]. *Dianthus* was set as a reference.

### PCR amplification of *infA* and *rpl23* genes

To detect the *infA* and *rpl23* genes, the genomic DNA of *Dianthus* was used as a template and the gene specific primers were designed with Primer3 v. 0.4.0 [37]. The *infA* and *rpl23* genes were amplified by PCR using gene specific primers (*infA*F: 5′-TGCGGATCAGACGACATTTT-3′ and *infA*R: 5′-GCAATTGGCGGAGAAATTTT-3′) and (*rpl23*F: 5′-TGCATTTCGATTAGGGTCGT-3′ and *rpl23*R: 5′-CAACGGAATCTCATCATCCA-3′) (S1 Fig). PCR products were purified using the Solg™ Gel & PCR Purification System Kit (Solgent Co., Daejeon, South Korea) according to the manufacturer’s protocols. Purified PCR products were sequenced with an ABI 3730XL DNA analyzer (Applied Biosystems, Foster City, CA, USA) at Solgent Co., South Korea. Other *infA* and *rpl23* genes of *Lychnis*, *Spinacia*, *Nicotiana*, *Solanum* and *Arabidopsis* were obtained from the NCBI database. All nucleotide sequences were aligned using MAFFT v7. 017 [38] in Genious v7.1.7 (Biomatters, New Zealand).

### Analysis of tandem repeats and single sequence repeats (SSR)

PHOBOS v3.3.12 was used for the detection of tandem repeats and single sequence repeats (SSR). The analysis parameters of alignment scores for the match, mismatch, gap, and N positions were set as 1, -5, -5, and 0, respectively [39].

### Analysis of RNA editing

The online program, Predictive RNA Editor for Plants (PREP) suite (http://prep.unl.edu/) [40], was used for the analysis of possible RNA editing sites in protein-coding genes of the *Dianthus cp genome*. For this analysis, the cut-off value was set at 0.8. The PREP-cp program has 35 reference genes for revealing RNA editing sites in the chloroplast genomes.

### Synonymous (K_S_) and nonsynonymous (K_A_) substitution rate analysis

The completed cp genome sequence of *Dianthus* was compared with the cp genome sequences of *Lychnis* and *Spinacia*. To analyze synonymous (K_S_) and nonsynonymous (K_A_) substitution rates, the same individual functional protein-coding exons were extracted and translated into protein sequences and aligned separately using Geneious v7.1.7. The synonymous (K_S_) and nonsynonymous (K_A_) substitution rates for each protein-coding exon were estimated in DnaSP [41].

### Phylogenetic analysis

The 31 completed cp genome sequences representing the lineages of angiosperms were downloaded from the NCBI Organelle Genome Resource database (S1 Table). The individual protein coding genes *infA*, *rpl23* and *clpP* from 32 angiosperms (including *Dianthus*) were analyzed and investigated separately for evolutionary gene significance. The nucleotide sequences of each gene were subjected to Geneious alignment using Geneious v7.1.7. The 78 protein-coding gene sequences and three individual sequences were aligned using MAFFT v7.017 [38] through Geneious v7.1.7 separately. The aligned protein-coding gene sequences were saved in PHYLIP format using Clustal X v2.1 [42] and used to generate a phylogenetic tree. Maximum likelihood (ML) analysis was performed with RaxML v7. 0 [43] using the general time-reversible invariant-sites (GTRI) nucleotide substitution model with the default parameters. The bootstrap probability of each branch was calculated by 1000 replications.

## Results

### Comparison of the *D*. *superbus* chloroplast genome organization and gene contents with other cp genomes

**Fig 1:**
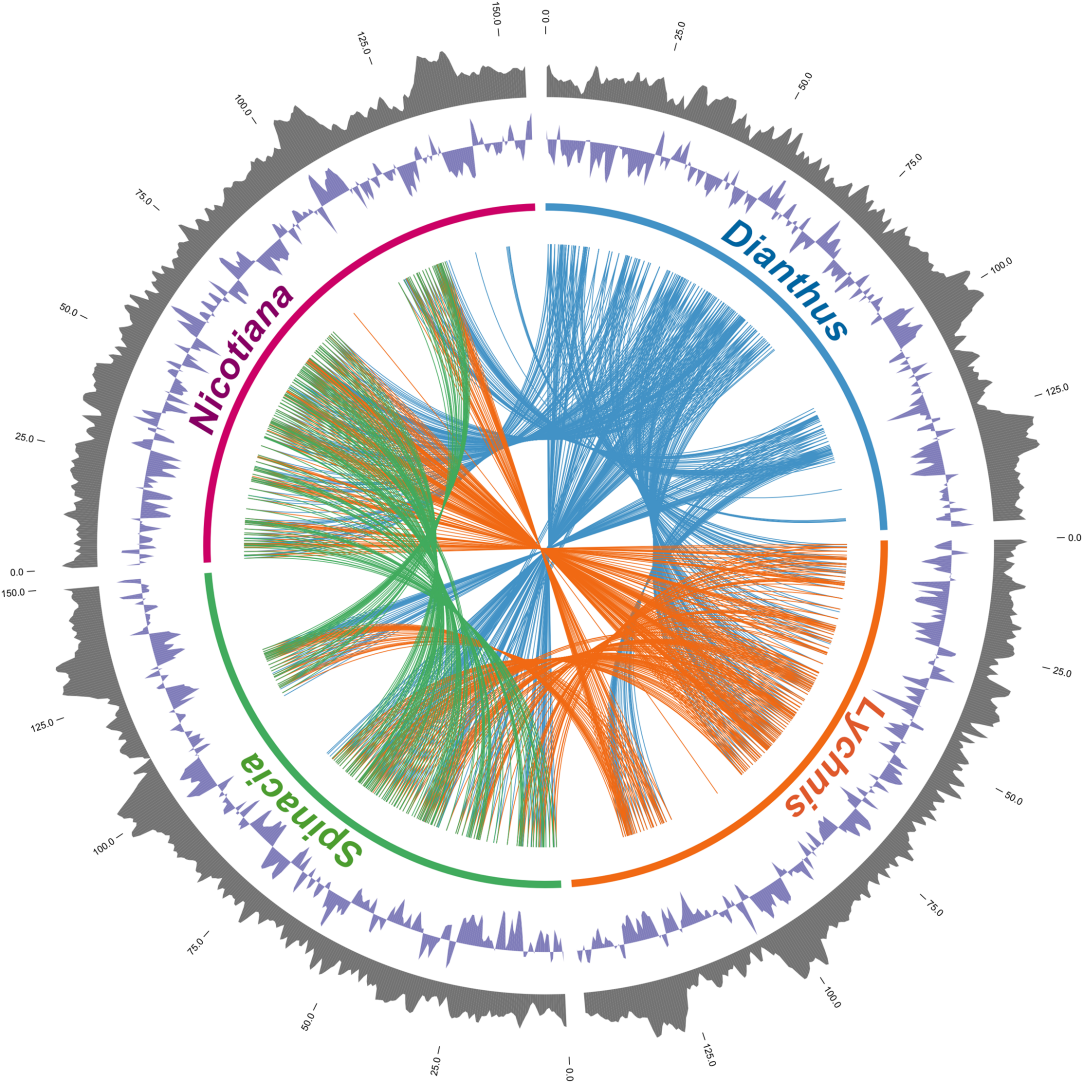
The circos diagram shows the four cp genomes. The outermost layer and inner layer denotes the % GC content and GC skew in the genome, respectively. The number of each genome indicates the genomic position on the assembly of chloroplast genomes. Similar blocks are connected with lines, and each line represents one unique gene of the genome with the highest similarity between *Dianthus* and three other cp genomes.

The cp genome of a medicinal plant, *D*. *superbus* var. *longicalycinus*, was analyzed, characterized and compared with its closely related species. The genome organization, gene content, GC skew and GC content of the four cp genomes were compared. The Circos diagram demonstrated a tightly genomic relationship between *Dianthus* and other cp genomes (Fig 1). The *Dianthus* cp genome encodes 78 protein coding genes, 30 tRNA genes, and four rRNA genes (Table 1). Seventeen genes are duplicated in the IR regions. The cp genome also has 17 intron-containing genes, 14 of which (8 protein-coding and 6 tRNA genes) are encoded in one intron and three (*clpP*, *rps12* and *ycf3*) that are encoded in two introns (Table 2). All genes had a common start codon (ATG) in the initiation site, except *rps19*, which carried ACG as a start codon.

**Table 1:** List of genes present in the *Dianthus* chloroplast genome.

Category	Gene group	Gene name				
Self-replication	Ribosomal RNA genes	*rrn4*.*5* ^a^	*rrn5* ^a^	*rrn16* ^a^	*rrn23* ^a^	
	Transfer RNA genes	*trnA*-UGC^a^ ^,^ ^b^	*trnC*-GCA	*trnD*-GUC	*trnE*-UUC	*trnF*-GAA
		*trnfM*-CAU	*trnG-*GCC	*trnG*-UCC^b^	*trnH*-GUG	*trnI*-CAU^a^
		*trnI*-GAU^a^ ^,^ ^b^	*trnK*-UUU^b^	*trnL*-CAA^a^	*trnL*-UAA^b^	*trnL*-UAG
		*trnM*-CAU	*trnN*-GUU^a^	*trnP*-UGG	*trnQ*-UUG	*trnR*-ACG^a^
		*trnR*-UCU	*trnS*-GCU	*trnS*-GGA	*trnS*-UGA	*trnT*-GGU
		*trnT*-UGU	*trnV*-GAC^a^	*trnV*-UAC^b^	*trnW*-CCA	*trnY*-GUA
	Small subunit of ribosome	*rps2*	*rps3*	*rps4*	*rps7* ^a^	*rps8*
		*rps11*	*rps12* ^a^ ^,^ ^c^ ^,^ ^d^	*rps14*	*rps15*	*rps16* ^b^
		*rps18*	*rps19*			
	Large subunit of ribosome	*rpl2* ^a^	*rpl14*	*rpl16* ^b^	*rpl20*	*rpl22*
		*rpl23* ^e^	*rpl32*	*rpl33*	*rpl36*	
	DNA-dependent RNA polymerase	*rpoA*	*rpoB*	*rpoC1* ^b^	*rpoC2*	
	Translational initiation factor	*infA* ^e^				
Genes for photosynthesis	Subunits of photosystem I	*psaA*	*psaB*	*psaC*	*psaI*	*psaJ*
		*ycf3* ^c^	*ycf4*			
	Subunits of photosystem II	*psbA*	*psbB*	*psbC*	*psbD*	*psbE*
		*psbF*	*psbH*	*psbI*	*psbJ*	*psbK*
		*psbL*	*psbM*	*psbN*	*psbT*	*psbZ*
	Subunits of cytochrome	*petA*	*petB* ^b^	*petD* ^b^	*petG*	*petL*
		*petN*				
	Subunits of ATP synthase	*atpA*	*atpB*	*atpE*	*atpF* ^b^	*atpH*
		*atpI*				
	Large subunit of Rubisco	*rbcL*				
	Subunits of NADH dehydrogenase	*ndhA* ^b^	*ndhB* ^a^ ^,^ ^b^	*ndhC*	*ndhD*	*ndhE*
		*ndhF*	*ndhG*	*ndhH*	*ndhI*	*ndhJ*
		*ndhK*				
Other genes	Maturase	*matK*				
	Envelope membrane protein	*cemA*				
	Subunit of acetyl-CoA	*accD*				
	C-type cytochrome synthesis gene	*ccsA*				
	Protease	*clpP* ^c^				
	Component of TIC complex	*ycf1* ^a^				

^a—^Two gene copies in IRs;

^b—^Gene containing a single intron;

^c^—Gene containing two introns;

^d^—Gene divided into two independent transcription units;

^e—^Pseudogene.

**Table 2:** Location and length of intron-containing genes in the *Dianthus* chloroplast genome.

Gene*	Location	Exon I	Intron I	Exon II	Intron II	Exon III
		Nucleotides in base pairs				
*atpF*	LSC	144	690	410		
*clpP*	LSC	69	857	291	567	228
*ndhA*	SSC	552	1049	540		
*ndhB*	IR	777	663	756		
*petB*	LSC	6	712	642		
*petD*	LSC	7	755	477		
*rps12* ^*#*^	LSC	114	--	232	538	26
*rpl16*	LSC	9	912	402		
*rpoC1*	LSC	432	736	1620		
*rps16*	LSC	40	837	227		
*trnG*-UCC	IR	38	808	35		
*trnA*-UGC	IR	23	695	48		
*trnI*-GAU	IR	42	913	35		
*trnK*-UUU	LSC	37	2424	35		
*trnL-*UAA	LSC	37	541	50		
*trnV-*UAC	LSC	39	605	37		
*ycf3*	LSC	129	756	228	809	153

*Identical duplicate gene containing introns in the IR region are not included.

^#^ The *rps12* is a trans-spliced gene with the 5′ end located in the LSC region and duplicated in the 3′ end in the IR regions.

Most of the genes were present in all cp genomes. The other Caryophyllales species, *Lychnis* and *Spinacia* and *Nicotiana*, also encode 30 tRNAs and four RNAs. Nevertheless, the Caryophyllales share an identical number of protein coding genes (78 genes), but *Nicotiana* encodes 88 protein coding genes. Intron containing genes varied among these species. Both *Dianthus* and *Spinacia* contain 17 intron containing genes, whereas *Lychnis* and *Nicotiana* have 16 and 15 intron genes, respectively. The value of *Dianthus* GC content is similar to that of *Lychnis* (36.3%), while that of *Spinacia* is 34.8% and *Nicotiana* 37.8% (Fig 1).

**Fig 2:**
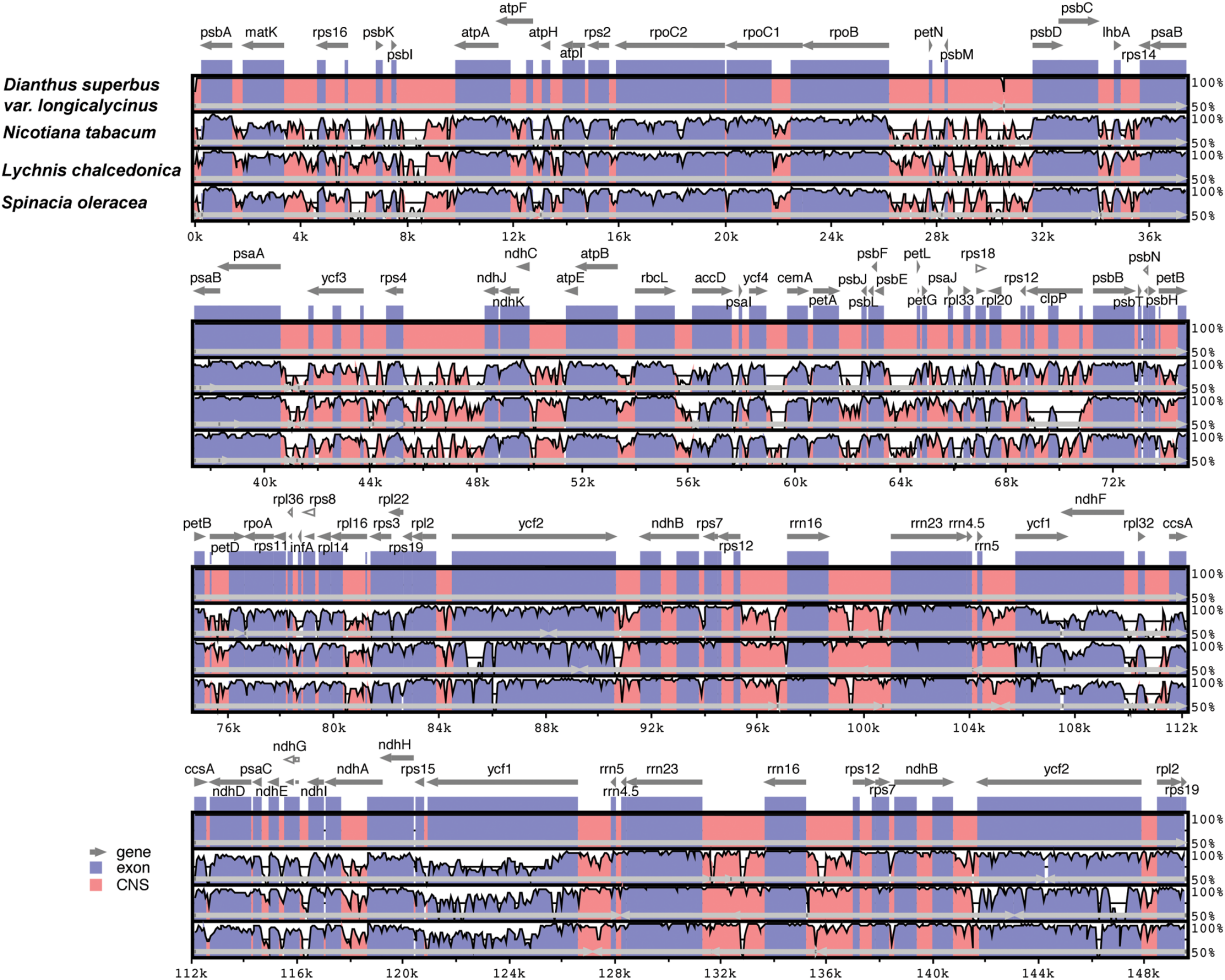
Comparison of the cp genome sequence of *Dianthus superbus* var. *longicalycinus*, *Nicotiana tabacum*, *Lychnis chalcedonica* and *Spinacia oleracea* generated with mVista. Gray arrows indicate the position and direction of each gene. Red and blue areas indicate intergenic and genic regions respectively. Black lines define regions of sequence identity with *D*. *superbus* var. *Longicalycinus*, using a 50% identity cutoff. Dashed rectangles denote highly divergent regions of *D*. *superbus* var. *longicalycinus* compared to *N*. *tabacum*, *L*. *chalcedonica* and *S*. *oleracea*.

mVISTA was employed to study sequence variations in the Caryophyllaceae family and *Nicotiana*. This analysis revealed that the coding region is more highly conserved than the non-coding regions (Fig 2). However, the most dissimilar coding regions of the four chloroplast genomes were *clpP*, *infA*, *ycf1* and *ycf2*.

### Comparisons of boundary regions of *Dianthus* with closely related cp genomes

**Fig 3:**
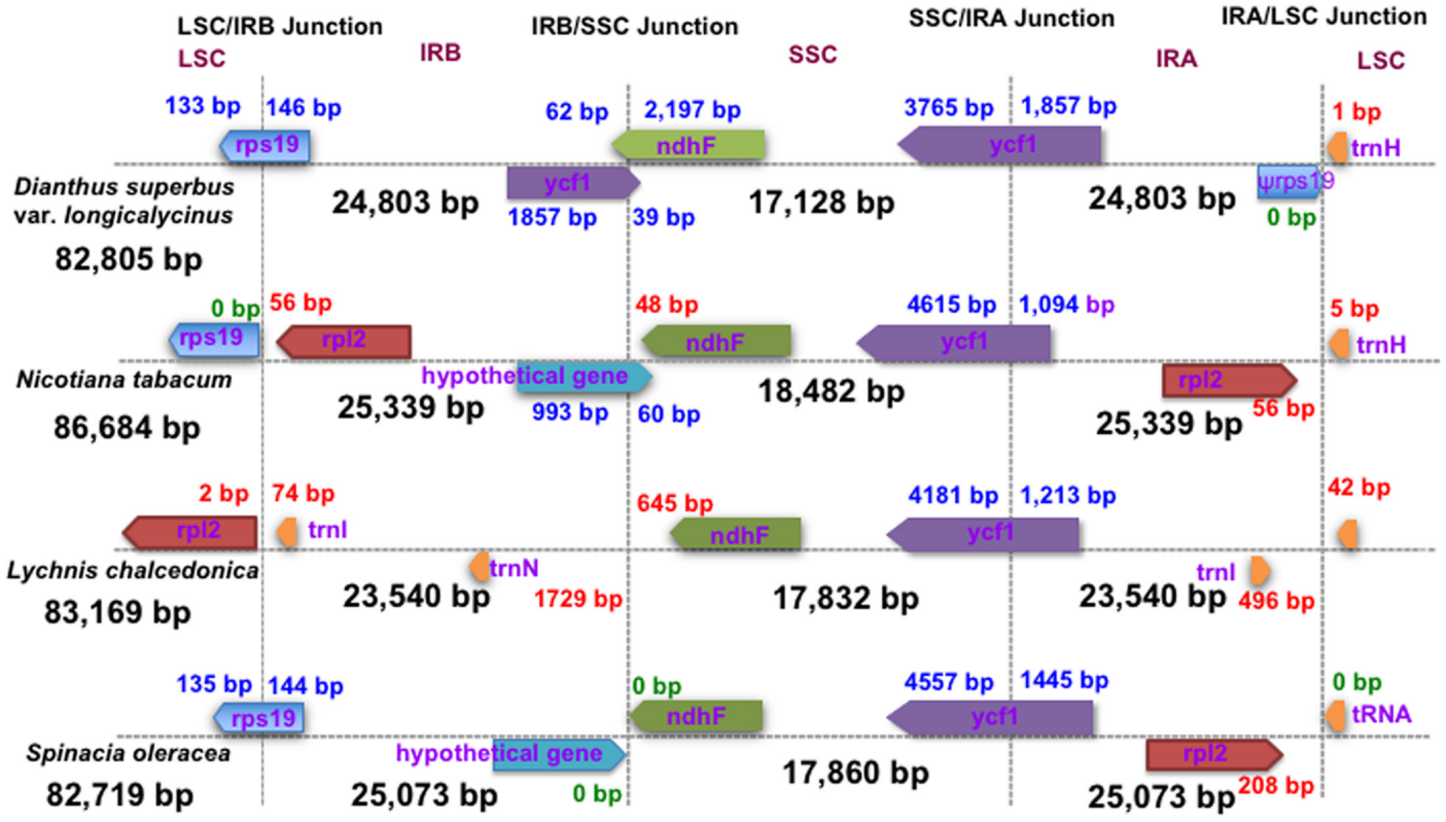
Comparison of the borders of the LSC, SSC and IR regions of *D*. *superbus* var. *longicalycinus*, *N*. *tabacum*, *L*. *chalcedonica* and *S*. *oleracea* cp genomes. (ψ refers to the pseudogene of *rps19* at the IRA/LSC border of *D*. *superbus* var. *longicalycinus*). Blue numbers indicate the amount of bp present and red indicates gaps between genes and junctions.

The LSC/IRB/SSC/IRA boundary regions of the *Dianthus* cp genome were compared to the corresponding regions of the three other cp genomes of *Lychnis*, *Spinacia* and *Nicotiana* (Fig 3). The *rps19* gene of *Dianthus* (133 bp of 279 bp) and *Spinacia* (135 bp of 279 bp) was extended from the IRB to the LSC region with 2 bp variability. However, the *rps19* gene of *Nicotiana* was shifted to an LSC region with a 2 bp gap and absent from *Lychnis*. At the IRB/SSC boundary, the *ycf1* and *ndhF* genes of *Dianthus* overlapped, whereas the *ycf1* gene of *Lychnis* was not present. Expansion, contraction and shifting of the *ycf1* gene was observed in the boundary regions of SSC/IRA. The size variation of *ycf1* from 5394 bp to 6002 bp was identified in all cp genomes. However, the pseudogene *rps19* was only present in the IRA/LSC junctions of the *Dianthus* genome. The *trnH* gene was located in the LSC region of all genomes, but varied from 1 bp to 42 bp apart from the IRA/LSC junctions. When compared with other closely related cp genomes of Caryophyllacaee, the IR region of *Dianthus* (24,803 bp) was found to be smaller than that of *Spinacia* (25,073 bp), but larger than the *Lychnis* IR region (23,540 bp).

### Pseudogenization of *infA* and *rpl23* genes

**Fig 4:**
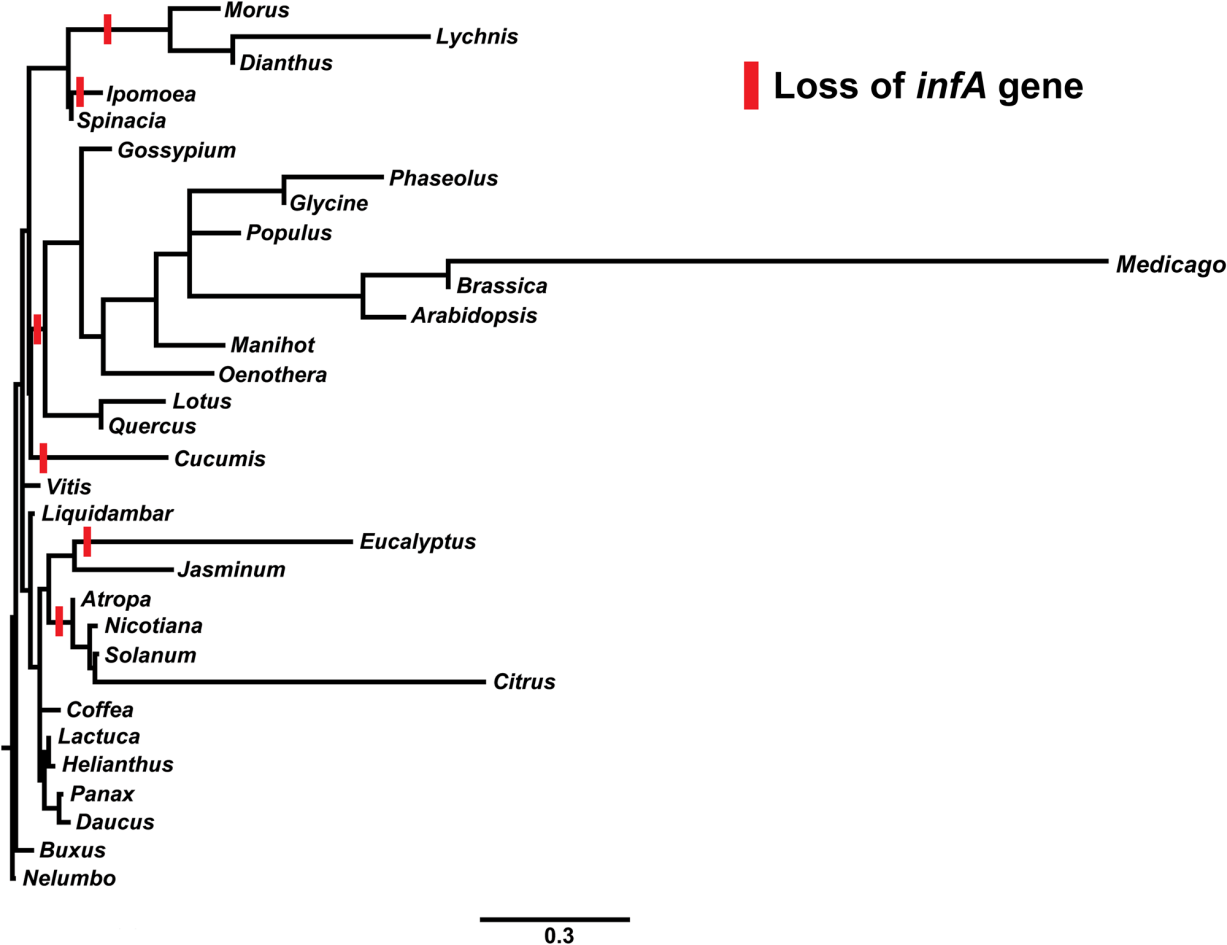
Molecular phylogenetic tree analysis of cp protein-coding gene *infA* of 32 Angiosperms. The tree was constructed by maximum likelihood (ML) analysis using the RaxML program and the GTR+I nucleotide model. The stability of each tree node was tested by bootstrap analysis with 1000 replicates. *Nelumbo* was set as the outgroup.

**Fig 5:**
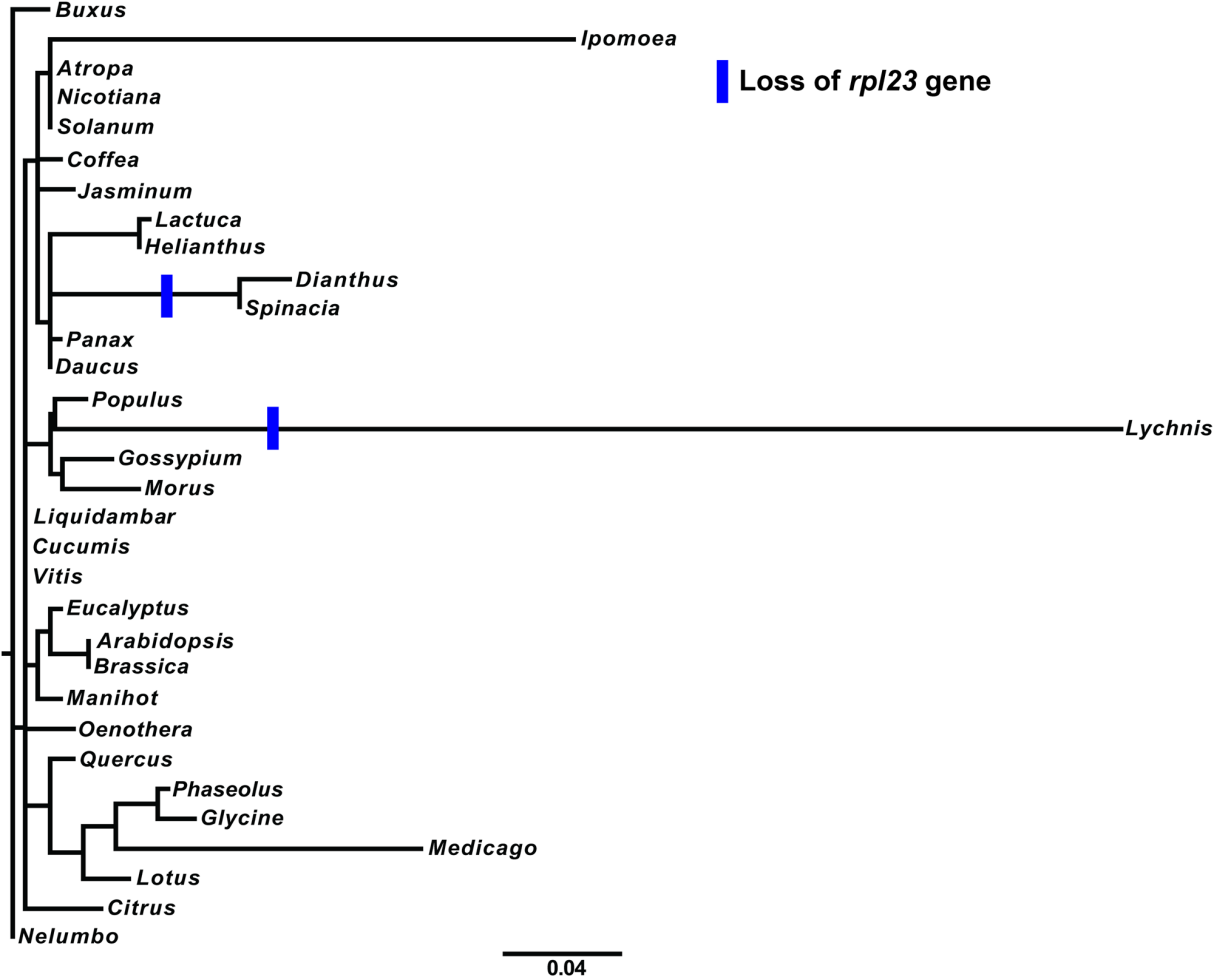
Molecular phylogenetic tree analysis of cp protein-coding gene *rpl23* of 32 Angiosperms. The tree was constructed by maximum likelihood (ML) analysis using the RaxML program and the GTR+I nucleotide model. The stability of each tree node was tested by bootstrap analysis with 1000 replicates. *Nelumbo* was set as the outgroup.

The chloroplast genes *infA* and *rpl23* of *Dianthus* were analyzed with 31 other angiosperms. Both *infA* and *rpl23* were found to be pseudogenes in the cp genome of *Dianthus*. Among 32 angiosperms (including *Dianthus*), the *infA* gene was found to be a pseudogene or entirely missing from *Dianthus* and *Lychnis* of the Caryophyllales family, as well as Brassicales, Cucurbitales, Fabales, Malpighiales, Malvales, Myrtales and Sapindales of Rosids and Solanales of Asterids (Fig 4 and S2 Fig). Comparative analysis of the ribosomal protein gene, *rpl23*, in 32 angiosperms revealed that it was a pseudogene or lost gene exclusively in members of the Caryophyllales family such as *Dianthus*, *Lychnis* and *Spinacia* (Fig 5 and S3 Fig).

### Repeat sequence analysis

The occurrence, type and distribution of simple sequence repeats (SSR) or microsatellites was analyzed in the cp genome of *Dianthus*. A total of 10,543 SSRs were identified (Table 3), among which homopolymers were most common, accounting for 95.58% of the SSRs, whereas di-, tri-, tetra-, penta- and hexa polymers occurred with less frequency. Of the homopolymers, the occurrence of A/T and G/C sequences was 73.7% and 21.88%, respectively. However, the presence of dipolymers was 3.56%, while that of tri- and tetra polymers was 0.99% and 0.11%, respectively. Moreover, only one penta- and hexa polymer was observed in the cp genome. The size and location of tetra-, penta- and hexapolymers are shown in Table 4. A total of 13 polymers were identified in the genome, whereas nine were localized in intergenic spacers, four in coding regions and none in introns.

**Table 3:** List of identified simple sequence repeats of the *Dianthus* chloroplast genome.

SSR sequence	Number of repeats																	
	3	4	5	6	7	8	9	10	11	12	13	14	15	16	17	18	19	Total
A/T	-					101	40	30	8	7	6	2	4	1	2	1	1	203
G/C						1	1	1										3
AC/GT	43	2																45
AG/CT	101	10																111
AT/AT	162	37	7	1	2													209
CG/CG	10																	10
AAC/GTT	9																	9
AAG/CTT	18																	18
AAT/ATT	25	6	2	1														34
ACC/GGT	4																	4
ACT/AGT	2																	2
AGC/GCT	5																	5
AGG/CCT	3																	3
ATC/GAT	2																	2
AAAC/GTTT	1																	1
AAAG/CTTT	2																	2
AAAT/ATTT	3	1																4
AAGG/CCTT	1																	1
AATC/GATT	1																	1
AATT/AATT	1																	1
ACCT/AGGT	2																	2
AATAC/GTATT	1																	1
AATGGG/CCCATT	1																	1
Total																		672

**Table 4:** Distribution of tetra, penta and hexapolymer single sequence repeats (SSRs) in the *Dianthus* chloroplast genome.

SSR type	SSR sequence	SSR size (bp)	Start	End	Location
tetra	(AAAG)3	12	57620	57631	*accD* (CDS)
tetra	(AAAT)3	12	45281	45292	*rps4*/*trnT*-UGU (IGS)
tetra	(AAAT)3	12	68400	68411	*rpl20*/*rps12* (IGS)
tetra	(AAAG)3	12	73744	73755	*psbH*/*petB* (IGS)
tetra	(AAAT)3	12	45232	45243	*rps4*/*trnT*-UGU (IGS)
tetra	(AAGG)3	12	130143	130154	*atpF*/*atpH* (IGS)
tetra	(AATC)3	12	114993	115004	*ndhE* (CDS)
tetra	(AAAC)3	12	45317	45328	*rps4*/*trnT*-UGU (IGS)
tetra	(ACCT)3	12	102711	102722	*rrn23* (CDS)
tetra	(AATT)3	12	47846	47857	*trnF*-GAA/*ndhJ* (IGS)
tetra	(AAAT)4	16	45916	45931	*trnT*-UGU/*trnL*-UAA (IGS)
penta	(AATAC)3	15	45410	45424	*rps4*/*trnT*-UGU (IGS)
hexa	(AATGGG)3	18	77126	77143	*rpoA* (CDS)

The distribution of tandem repeats with more than 20 bp and 100% sequence identity was also analyzed. The results revealed 19 tandem repeats in the cp genome of *Dianthus* (Table 5). Of these, 16 were found in the intergenic spacers of *trnE*-UUC/*trnT*-GGU (2), *trnT*-GGU (1), *psaA*/*ycf3* (1), *rps4*/*trnT*-UGU (3), *trnT*-UGU/*trnL*-UAA (1), *atpB*/*rbcL* (1), *rbcL*/*accD* (2), *trnP*-UGG/*psaJ* (1), *clpP*/*psbB* (1), *rpl22*/*rps19* (1), *rpl32*/*trnL*-UAG (1) and *trnL*-UAG/*ccsA* (1) and three were situated in the intron sequence of *trnL*-UAA (1), *rpl16* (1) and *ndhA* (1). No tandem repeats were identified in the protein-coding regions.

**Table 5:** Distribution of tandem repeats in the *Dianthus* chloroplast genome.

S.No.	Repeat length (bp)	Consensus size × copy number	Start	End	Location
1	24	12×2	29889	29912	*trnE*-UUC/*trnT*-GGU (IGS)
2	24	12×2	30419	30430	*trnE*-UUC/*trnT*-GGU (IGS)
3	20	10×2	30672	30691	*trnT*-GGU/*psbD* (IGS)
4	22	11×2	41328	41349	*psaA*/*ycf3* (IGS)
5	22	11×2	45225	45246	*rps4*/*trnT*-UGU (IGS)
6	20	10×2	45631	45650	*rps4*/*trnT*-UGU (IGS)
7	20	10×2	45648	45667	*rps4*/*trnT*-UGU (IGS)
8	30	15×2	46377	46406	*trnT*-UGU/*trnL*-UAA (IGS)
9	22	11×2	46866	46887	*trnL*-UAA (Intron)
10	20	10×2	53295	53314	*atpB*-*rbcL* (IGS)
11	20	10×2	55592	55611	*rbcL*-*accD* (IGS)
12	20	10×2	55607	55627	*rbcL*-*accD* (IGS)
13	24	12×2	65797	65820	*trnP*-UGG/*psaJ* (IGS)
14	38	19×2	71150	71187	*clpP*/*psbB* (IGS)
15	44	22×2	80513	80556	*rpl16* (Intron)
16	20	10×2	82636	82655	*rpl22*/*rps19* (IGS)
17	20	10×2	110817	110836	*rpl32*/*trnL*-UAG (IGS)
18	46	23×2	111417	111462	*trnL*-UAG/*ccsA* (IGS)
19	22	11×2	118342	118342	*ndhA* (Intron)

### RNA editing

The PREP-cp program predicted 45 RNA editing sites in 16 genes of the *Dianthus* cp genome. Of these 16 genes, *ndhB* and *ndhD* encoded 10 RNA editing sites. The RNA editing types in *Dianthus* were all non-silent, and 100% C to U (S2 Table). Of these, 75.56% (34) occurred in the second base position of the codon, whereas 24.44% (11) were in the first position of the codon. The amino acid was changed due to nucleotide substitution in the codon. Among the 45 amino acids, 22 amino were converted from hydrophilic to hydrophobic (S to L, S to F and T to I), 12 from hydrophobic to hydrophobic (A to V, P to L and L to F), seven from hydrophilic to hydrophilic (T to M, H to Y and R to W) and four from hydrophobic to hydrophilic (P to S). Among these, 15 amino acids (33.3%) were converted from Serine to Leucine.

### Synonymous (K_S_) and nonsynonymous (K_A_) substitution rate analysis

**Fig 6:**
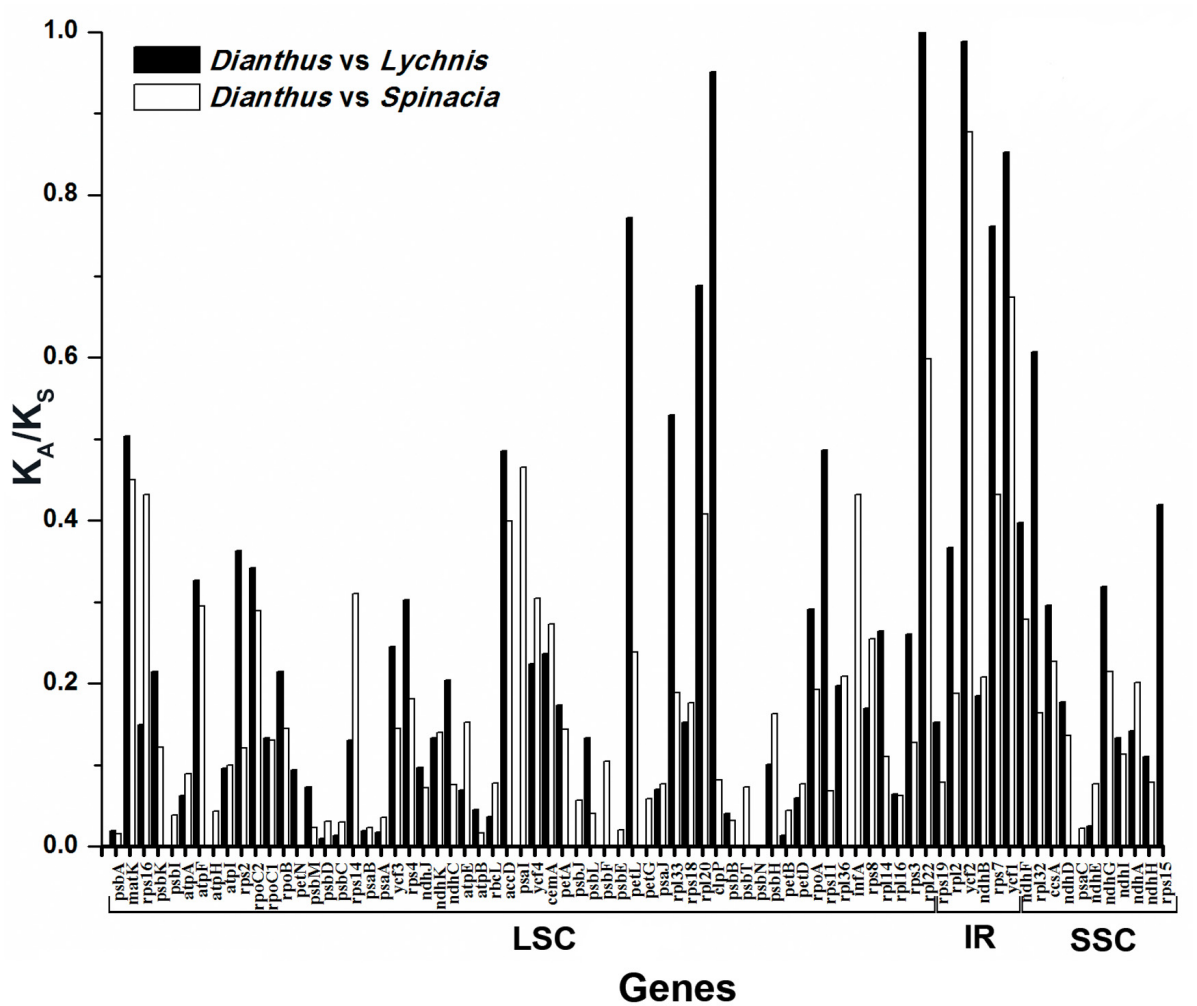
The K_A_/K_S_ values of 76 genes encoded by 87 protein-coding exons of *D*. *superbus* var. *longicalycinus*, *L*. *chalcedonica* and *S*. *oleracea*. Solid black and open boxes indicate the K_A_/K_S_ ratio of the *D*. *superbus* var. *longicalycinus* vs. *L*. *chalcedonica* and *D*. *superbus* var. *longicalycinus* vs. *S*. *oleracea*.

A total of 76 genes encoding 87 protein-coding exons in the cp genome of *Dianthus* were used to analyze synonymous and nonsynonymous rates against *Lychnis* and *Spinacia* (Fig 6). The K_A_/K_S_ ratio of all genes was less than 1, except for *rpl22* of *Lychnis*. The K_A_/K_S_ ratios of *rpl22* and *ycf2* of *Dianthus* vs. *Lychnis* were 1.03407 and 0.98866, respectively.

### Phylogenetic analysis

**Fig 7:**
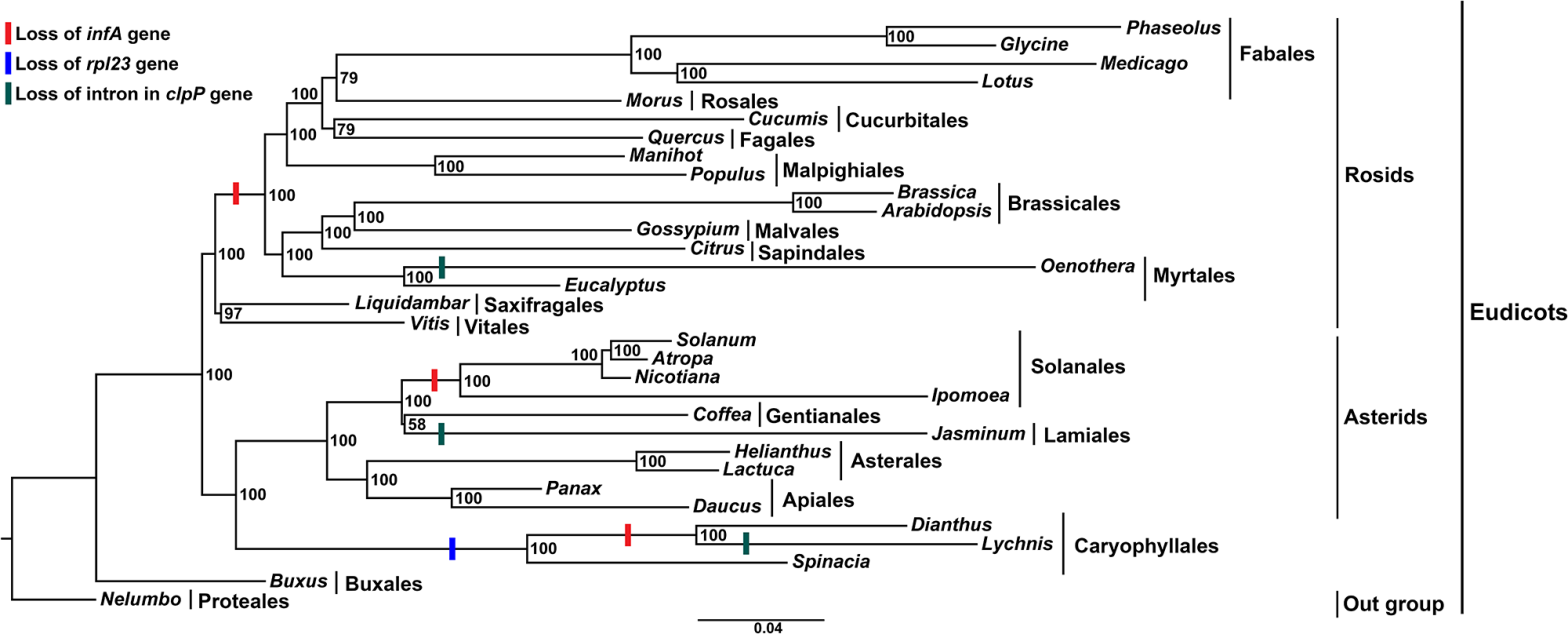
Molecular phylogenetic tree of 32 Angiosperms based on 78 protein-coding genes in the cp genome. The tree was constructed by maximum likelihood (ML) analysis of the conserved regions using the RaxML program and the GTR+I nucleotide model. The stability of each tree node was tested by bootstrap analysis with 1000 replicates. Bootstrap values are indicated on the branches, and the branch length reflects the estimated number of substitutions per 1000 sites. *Nelumbo* was set as the outgroup.

A molecular phylogenetic tree was constructed using 78 protein coding genes of 32 cp genome sequences. Among these 32 taxa, *Nelumbo* was set as the outgroup. The phylogenetic tree was divided into two clades, rosids and asterids. Within asterids, Caryophyllales (core eudicots) diverged from asterids and formed two sister clades with a 100% bootstrap (BS) value. The Caryophyllales contained two sub sister clades. The first sub clade included *Spinacia* (Amaranthaceae), whereas *Dianthus* and *Lychnis* (Caryophyllaceae) were in the second sub clade with a 100% BS value (Fig 7).

## Discussion

A medicinal plant, *D*. *superbus* var. *longicalycinus* cp, was characterized and compared to closely related species by comparative genome analysis. The cp genomes of Caryophyllaceae family plants contained 78–82 protein encoding genes and 45 RNA genes. However, the *Dianthus* cp genome had 78 protein-encoding genes and 34 RNA genes. The total number of proteins encoded by protein-coding genes of the *Dianthus* cp genome was found to be greater when compared to other Caryophyllaceae plants; however, it was the fourth smallest of the nine completed Caryophyllaceae cp genomes (after including *D*. *superbus*). The *Dianthus* cp genome was larger than that of *Silene Conica* (1,47,208 bp), *S*. *conoidea* (1,47896 bp) and *L*. *chalcedonica* (1,48,081 bp), but smaller than the cp genomes of *S*. *vulgaris* (1.515 Kb), *S*. *paradoxa* (1.516 Kb), *S*. *noctiflora* (1.516 Kb), *S*. *latifolia* (1.517 Kb) and *Agrostemma githago* (1.517 Kb). When compared with other Caryophyllaceae cp genomes, *Dianthus* had the smallest LSC (82,805 bp) and SSC regions (17,128 bp).

Comparative genome analysis revealed several dissimilarities in the Caryophyllaceae family. Comparison of the contents of *Dianthus* with the other three cp genomes revealed that the protein coding, tRNA and rRNA regions were similar to those of *Lychnis* and *Spinacia*, encoding 78, 30 and 4 genes, respectively. This might have been because the genome shares its gene contents with the Caryophyllales family. However, the total number of introns in the plastid differs within this family. Specifically, *Dianthus* and *Spinacia* share a total of 22 introns in the cp genome, whereas *Lychnis* contains only 20 introns. This was due to the loss of two introns in the *clpP* gene of *Lychnis*. This intron loss might have been due to the rapidly evolving *clpP* gene in the *Lychnis* species [32,44]. Conversely, *Nicotiana* contains 24 introns in the cp genome [23]. The difference in the intron between *Nicotiana* and *Caryophyllacea* was due to the absence of an intron in the *rpl2* gene of Caryophyllales. Downie *et al*. [45] revealed that several lineages of flowering plants had lost introns from the *rpl2* gene independently, which could also be considered a distinguishing feature of core members of Caryophyllales [46].

The occurrence of IR regions could help stabilize the cp genome, and the most significant feature of the IR region is its resistance to recombinational loss [47]. Goulding *et al*. [48] reported that fluctuations have occurred sporadically in the IR regions of Angiosperms during evolution. A copy of IR genes was lost during the rearrangement of cpDNA evolution of Angiosperms [49]. As shown in Fig 3, the IRs have both extended and constricted during evolution of the Caryophyllacea family plants; however, intense variations were not identified. Nevertheless, some variations were detected in the IRA/LSC regions. Some species encoded two copies of the *rps19* gene near the IRB/SSC and IRA/LSC junctions, while the *Dianthus* cp genome encoded one copy of the *rps19* gene at the IRB/SSC junction and the pseudogene *rps19* was observed at the IRA/LSC junction. The length of the pseudogene *rps19* was shorter (146 bp) than that of the regular *rps19* gene (279 bp). This pseudogenization might have been due to IR fluctuation in the cp genome of *Dianthus*. Interestingly, the ACG start codon was found in *rps19*. Neckermann *et al*. [50] reported that the ACG start codon has been converted into an initiation codon, AUG, in *Nicotiana* due to RNA editing in the translation process. This might also have occurred in the *D*. *superbus* var. *longicalycinus* cp genome. Taken together, this evidence indicates that evolutionary rates of cp genomes in the Caryophyllaceae are comparatively mild based on the relatively minor variations in the IR regions.

The *infA* and *rpl23* genes appeared as pseudogenes or were lost from the cp genome of *Dianthus*. The functional gene sequence of *infA* was highly variable in Caryophyllales. The *infA* gene of *Dianthus* differed from that of other Caryophyllales such as *Spinacia* and asterids (*Coffee*, *Daucus*, *Helianthus*, *Jasminum*, *Lactuca* and *Panax*) and Rosids (*Liquidambar* and *Vitis*) because of the presence of a pseudogene, *infA*, in *Dianthus* and *Lychnis* (Fig 7). However, *Spinacia* encodes a functional intact *infA* gene in the Caryophyllales family. When compared with the other cp genome of *Spinacia*, 170 bp of the *infA* gene were deleted from *Dianthus*, possibly due to a double frameshift mutation (6 bp insert) near the 3′ end. Previous studies also suggested that a 124 bp deletion occurred in the *infA* gene of tomato [18]. Earlier studies revealed that the *infA* gene was lost independently from multiple angiosperm lineages, including other species within the Caryophyllales [18,46,51,52]. Interestingly, another gene, *rpl23*, appears as a pseudogene or was lost from Caryophyllales. Earlier studies also suggested that both genes have been lost or subjected to pseudogenation in other Caryophyllales, including *S*. *latifolia*, *S*. *vulgaris*, *S*. *noctiflora*, *S*. *conica* and *Spinacia* [32,53]. Inversions, intron losses and substitution rate accelerations occurred independently in the cp genome of *L*. *chalcedonica* and *S*. *paradoxa* [32]. This gene loss might have been due to disruption of the nuclear-encoded DNA replication, recombination and repair machinery that regulates the cp genome [54]. These inversions and intron losses can be attributed to evolution of the plant organelle genome.

Further evolution of the *infA* and *rpl23* pseudogenes and intron containing gene, *clpP*, of *Dianthus* were compared with 31 other angiosperms. The gene and intron losses of different families formed a clade in the phylogenetic analysis that revealed that independent evolutionary lineages occurred in all three genes (Figs 4 and 5 and S4 Fig). The cp genes *chlB*, *chlL* and *chlN* have been lost independently from Gnetales and Gnetum [55] and *Welwitschia* [56]. The *infA* gene in *Ipomoea* and the *rps16* gene in *Passiflora* and *Populus* have also been lost independently [57]. Moreover, the *infA* and *rpl23* genes have been lost or pseudogenization occurred independently in the cp genome of *Dianthus*. However, parallel evolution occurred in the cp genome of *Lychnis* because of loss of the intron from the *clpP* gene [32]. Moreover, the intron loss of the *ClpP* gene has been indentified in *Cicer arietinum*, Poceae, Onagraceae, Oleaceae and Pinus [57,58]. Ronny *et al*. [18] also reported that cp *infA* was lost repeatedly during angiosperm evolution. The cp pseudogene, *rpl23*, in spinach has been functionally replaced by a nuclear gene, which is similar to the homologous cytosolic ribosomal protein gene [59]. Earlier studies reported that the genes responsible for ribosomal proteins or other translocation components are involved in gene loss in both the chloroplast and mitochondria genomes [60,61]. It includes the transfer of chloroplast genes *infA* and *rpl22*, substitution of chloroplast genes *rpl21* and *rpl23* and uncharacterized losses of several mitochondrial ribosomal protein genes in addition to the transfer of *rps10* [60,61].

Although chloroplast genomes are considered highly conserved regions in land plants, these regions with high sequence polymorphisms are frequently observed in closely related species [62]. The presence of several SSR sites in the cp genome of *Dianthus superbus* revealed that these sites can be evaluated for the intraspecific level of polymorphism, leading to highly sensitive phylogeographic and population structure studies for this species.

RNA editing is a post transcriptional process that has mainly occurred in mitochondrial and cp genomes of higher plants [63]. This process may induce substitution or indel mutations that lead to alternations in the process of transcription [9,63–65]. However, in the *ndhD* gene, the initiation codon, ACG, was altered to AUG by this editing process. RNA editing of C to U substitution has commonly occurred in most of the angiosperms [66], and the total number of editing sites varied from 20 to 37 [63,67–70]. However, comparison with other Caryophyllaceae family members such as *Lychnis* (48 editing sites) and *Spinacia* (47 editing sites) showed that the RNA editing sites and editing characteristics of *Dianthus* were similar. Chen *et al*. [63] also reported that closely related taxa generally share more RNA editing sites due to evolutionary conservation.

The nucleotide substitution patterns of synonymous and nonsynonymous are important indicators in gene evolution studies [71]. Makalowski and Boguski [72] reported that nonsynonymous substitutions occurred less frequently than synonymous substitutions, and the ratio of K_A_/K_S_ was less than one in most of the protein-coding regions. In this study, the ratio of K_A_/K_S_ was significantly less than one in all protein-coding regions of *Dianthus*. Nevertheless, the K_A_/K_S_ ratio of *rpl22* was 1.03407. This small fluctuation might have been due to nonsynonymous substitution in the *rpl22* gene and is the result of silent mutation. However, the *rpl22* nucleotide identity was less than 70% (66.6%) when compared with *Lychnis*.

Few studies have been conducted to analyze the phylogenetic relationships within the Caryophyllaceae family, and the phylogenetic evolution of *D*. *superbus* has yet to be investigated. Cuenoud *et al*. [73] reported that Caryophyllaceae was a sister clade to Amaranthaceae based on *matK* analysis. Clement *et al*. [74] revealed that anothocyanin pigment producing Caryophyllaceae was associated with betalain pigment producing Amaranthaceae. Our results also strongly supported that *Dianthus* (Caryophyllaceae) formed a sister clade to *Spinacia* (Amaranthaceae) with 100% BS value. Additionally, phylogenetic analysis strongly supports the loss or formation of a pseudogene of *infA* and *rpl23* in the cp genome of *Dianthus* (Fig 7). Because of the loss or absence of the *rpl23* gene from Caryophyllales, the clade diverged from asterids into a new separate clade. Another functional gene, *infA*, was lost from many angiosperms of land plants, including *Dianthus*. Owing to the absence or loss of the *infA* gene from *Dianthus* and *Lychnis*, *Spinacia* diverged from this clade and formed a subclade. When we investigated the evolutionary perspective of these genes, the *infA* and *rpl23* gene losses of different families were found to form a clade, which suggested that the evolutionary lineages have occurred independently.

## Conclusion

In summary, the *Dianthus* genome shares the same overall organization and gene contents of other cp genomes of Caryophyllaceae family members. However, several unique features were observed in the cp genome of *Dianthus*, including pseudogenization or gene loss of *rps19*, *infA* and *rpl23* genes. When compared with the other 31 angiosperm lineages, the *infA* gene has been lost from most members of the rosids, solanales of asterids and *Lychnis* of Caryophyllales, whereas the *rpl23* gene was lost or pseudogization has occurred exclusively in the family of Caryophyllales cp genomes. Phylogenetic analysis of individual protein-coding genes *infA* and *rpl23* has also revealed that gene loss or pseudogenization occurred independently in the cp genome of *Dianthus*. Molecular phylogenetic analysis of 78 protein-coding genes revealed that *Dianthus* is most closely related to *Lychnis* and *Spinacia*. Overall, the results of this study will contribute to a better understanding of the evolution, molecular biology and genetic improvement of the medicinal and ornamental plant, *D*. *superbus* var. *longicalycinus*.

## Supporting Information

S1 Fig PCR amplification of *rpl36*-*rps8* (*infA*) and *rpl2*-*trnI*-GAU (*rpl23*) nucleotide regions of *Dianthus* cp genome. Lane M: 1 kb BioFACT Plus Ladder; Lane 1: The *rpl36*-*rps8* region (535 bp); Lane 2: The *rpl2*-*trnI*-GAU region (675 bp).
(TIF)

S2 Fig Comparison of the *rpl36*-*rps8* region between *Dianthus superbus* var. *longicalycinus*, *Lychnis chalcedonica*, *Spinacia oleracea*, *Nicotiana tabacum*, *Solanum tuberosum* and *Arabidopsis thaliana* cp genomes. (TIF)

S3 Fig Comparison of the *rpl23* region between *Dianthus superbus* var. *longicalycinus*, *Lychnis chalcedonica*, *Spinacia oleracea* and *Nicotiana tabacum* cp genomes. (TIF)

S4 Fig Molecular phylogenetic tree analysis of cp protein-coding gene *clpP* of 32 Angiosperms. The tree was constructed by maximum likelihood (ML) analysis using the RaxML program and the GTR+I nucleotide model. The stability of each tree node was tested by bootstrap analysis with 1000 replicates. *Nelumbo* was set as the outgroup.
(TIF)

S1 Table Accession numbers of the chloroplast genome sequences used in this study. (DOCX)

S2 Table Prediction of RNA editing by the PREP-cp program. (DOCX)

S3 Table The nucleotide sequences of *rpl36*-*rps8* and *rpl23* regions of *Dianthus* cp genome. (DOCX)
