# Supplementary figures and images for: Analysis of the Complete Chloroplast Genome of a Medicinal Plant, Dianthus superbus var. longicalyncinus, from a Comparative Genomics Perspective
Source: PLoS One. 2015 Oct 29;10(10):e0141329. doi: 10.1371/journal.pone.0141329 (PMC4626046; doi:10.1371/journal.pone.0141329)

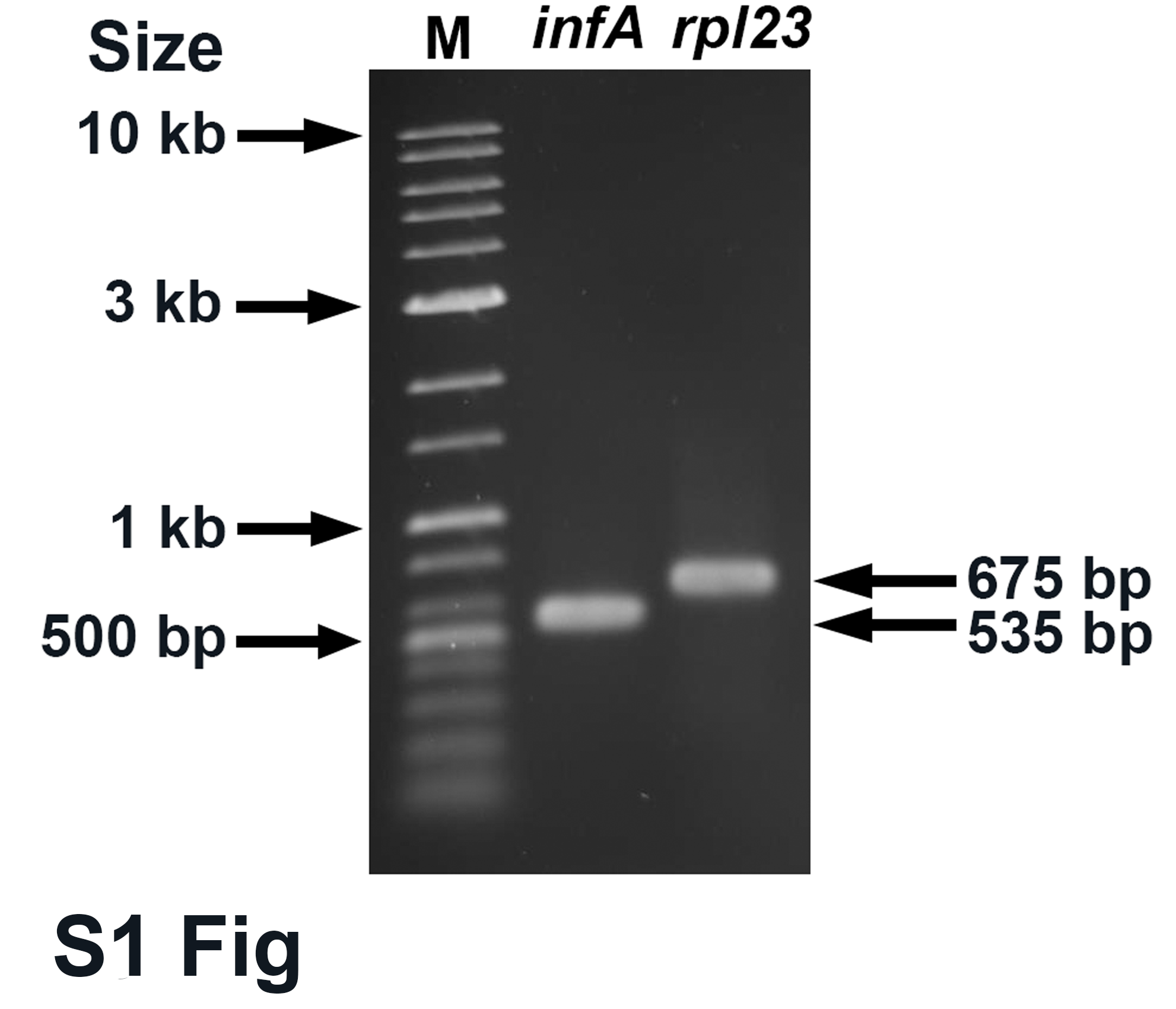

Supplement: S1 Fig — Lane M: 1 kb BioFACT Plus Ladder; Lane 1: The rpl36-rps8 region (535 bp); Lane 2: The rpl2-trnI-GAU region (675 bp). (TIF) [file pone.0141329.s001.tif]

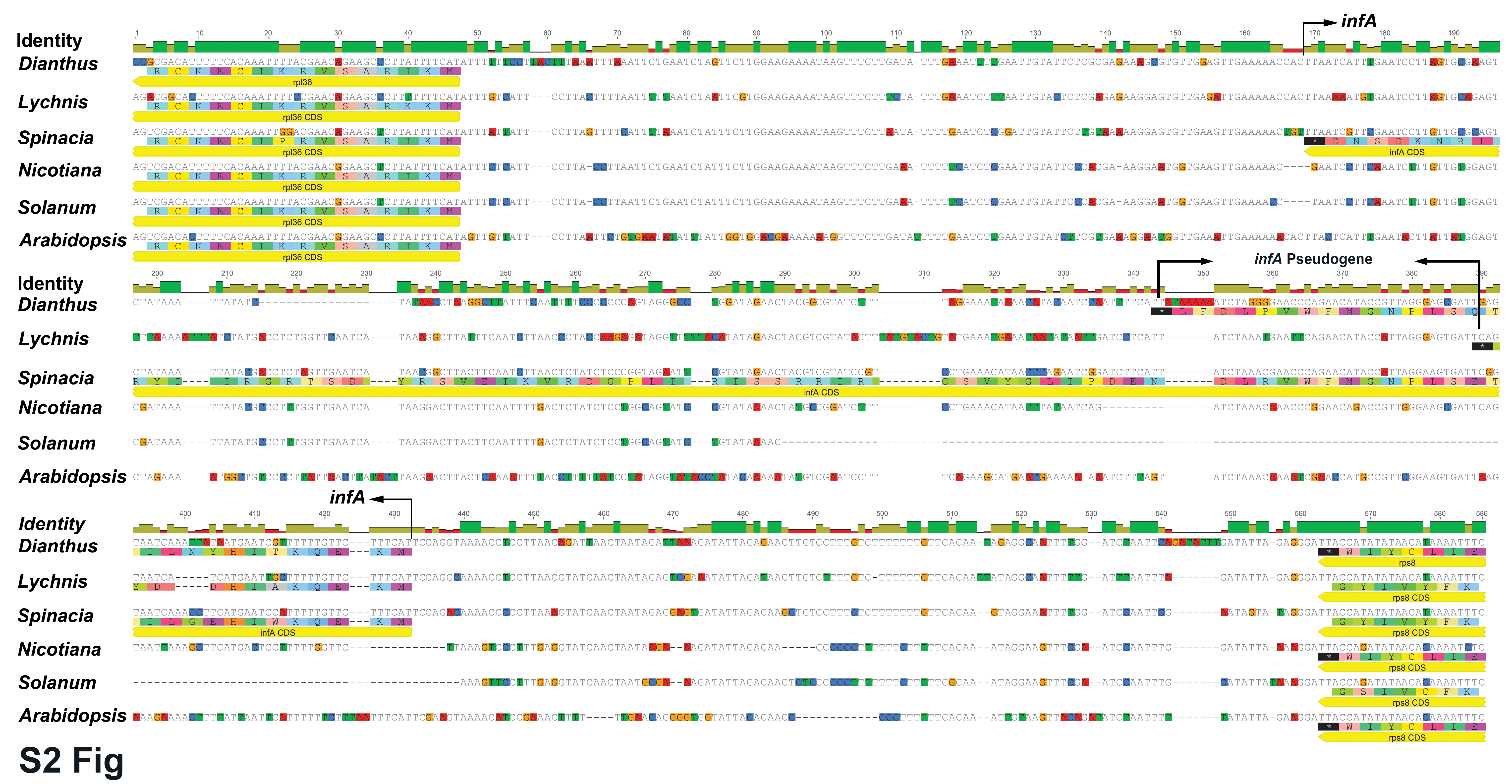

Supplement: S2 Fig — (TIF) [file pone.0141329.s002.tif]

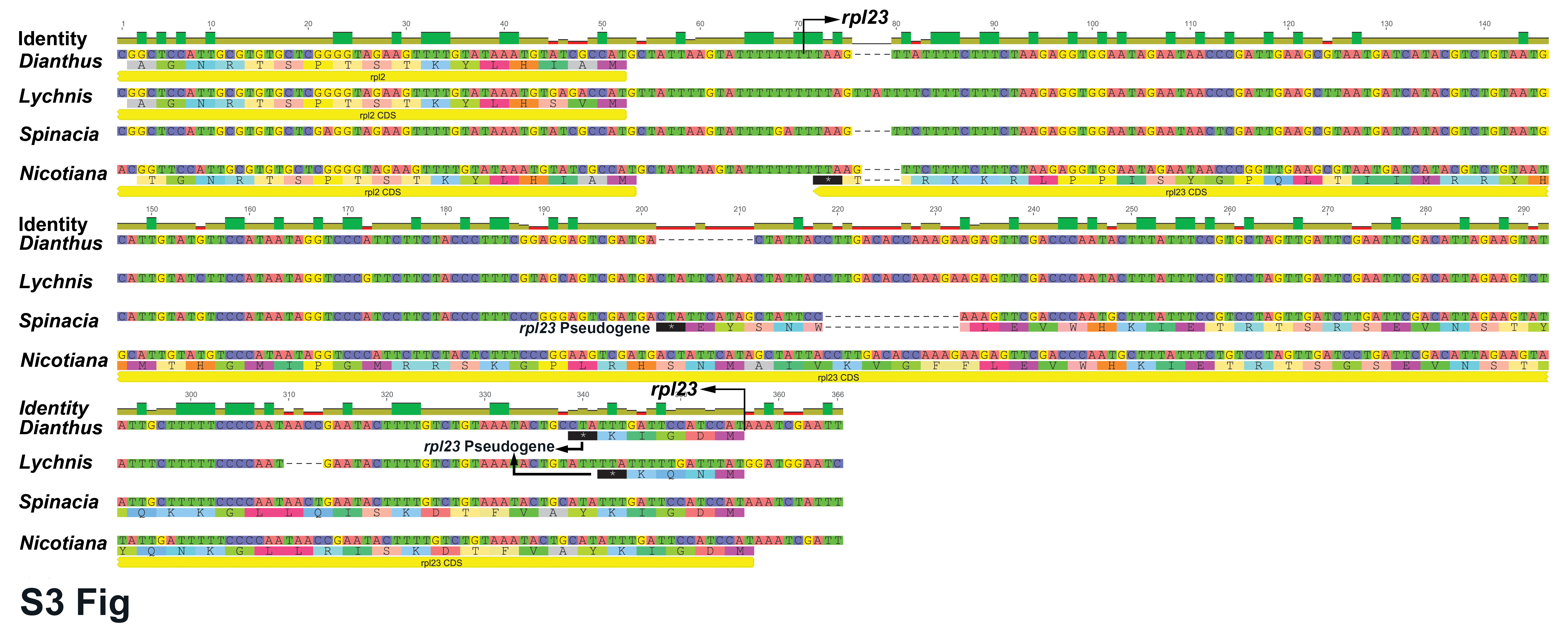

Supplement: S3 Fig — (TIF) [file pone.0141329.s003.tif]

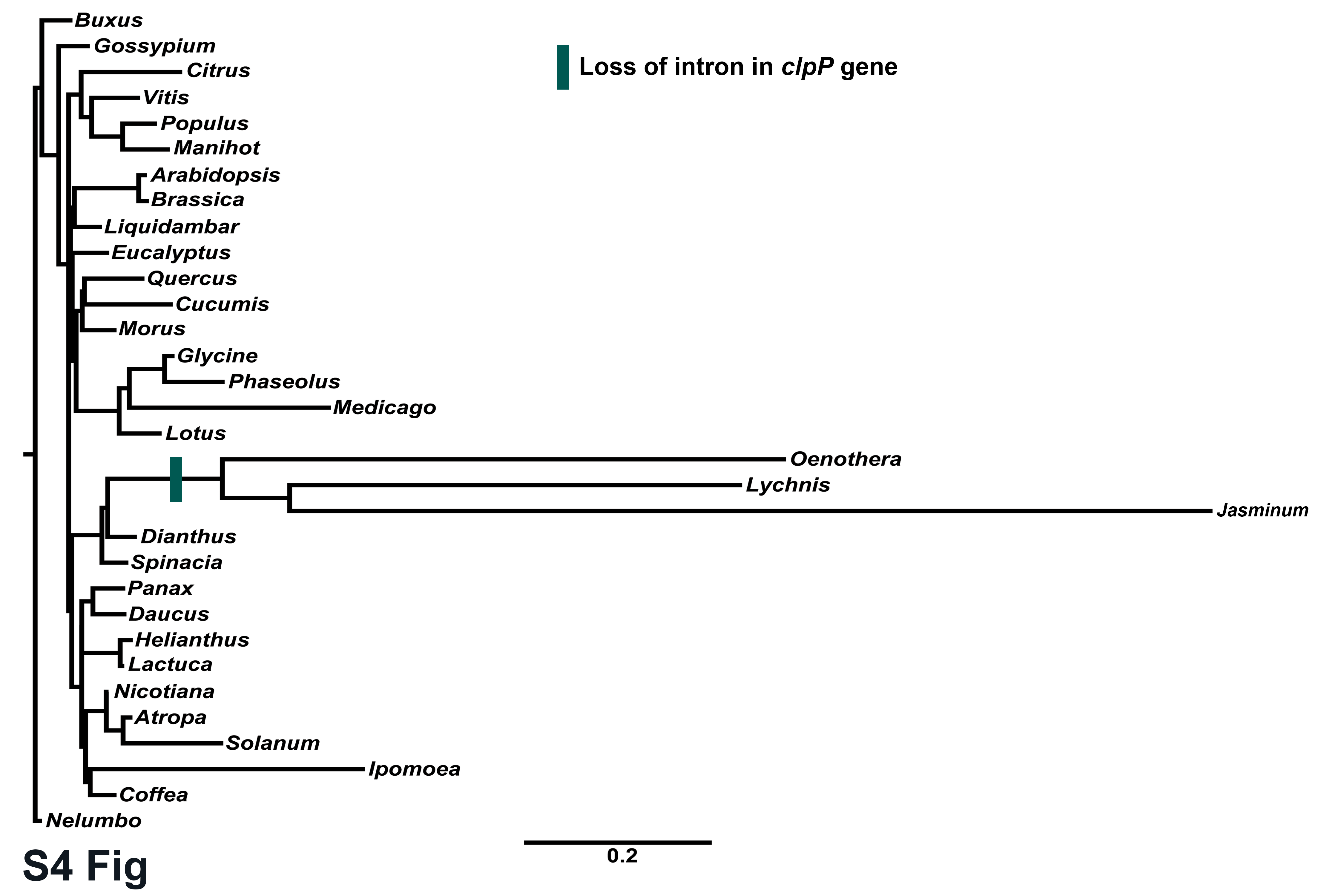

Supplement: S4 Fig — The tree was constructed by maximum likelihood (ML) analysis using the RaxML program and the GTR+I nucleotide model. The stability of each tree node was tested by bootstrap analysis with 1000 replicates. Nelumbo was set as the outgroup. (TIF) [file pone.0141329.s004.tif]
