# Supplementary material for: Analysis of the Complete Chloroplast Genome of a Medicinal Plant, Dianthus superbus var. longicalyncinus, from a Comparative Genomics Perspective
Source: PLoS One. 2015 Oct 29;10(10):e0141329. doi: 10.1371/journal.pone.0141329 (PMC4626046; doi:10.1371/journal.pone.0141329)
Supplement: S1 Table — (DOCX) [file pone.0141329.s005.docx]

**S1 Table. Accession numbers of the chloroplast genome sequences used in this study.**

| Sl. No. | Taxon | Family | Order | GenBank accession number |
| --- | --- | --- | --- | --- |
| 1 | *Arabidopsis thaliana* | Brassicaceae | Brassicales | NC_000932 |
| 2 | *Atropa belladonna* | Solanaceae | Solanales | NC_004561 |
| 3 | *Brassica napus* | Brassicaceae | Brassicales | NC_016734 |
| 4 | *Buxus microphylla* | Buxaceae | Buxales | NC_009599 |
| 5 | *Citrus sinensis* | Rutaceae | Sapindales | NC_008334 |
| 6 | *Coffea arabica* | Rubiaceae | Gentianales | NC_008535 |
| 7 | *Cucumis sativus* | Cucurbitaceae | Cucurbitales | NC_007144 |
| 8 | *Daucus carota* | Apiaceae | Apiales | NC_008325 |
| 9 | *Dianthus superbus* | Caryophyllaceae | Caryophyllales | KM668208 |
| 10 | *Eucalyptus camaldulensis* | Myrtaceae | Myrtales | NC_022398 |
| 11 | *Glycine max* | Fabaceae | Fabales | NC_007942 |
| 12 | *Gossypium barbadense* | Malvaceae | Malvaceae | NC_008641 |
| 13 | *Helianthus annuus* | Asteraceae | Asterales | NC_007977 |
| 14 | *Ipomoea purpurea* | Convolvulaceae | Solanales | NC_009808 |
| 15 | *Jasminum nudiflorum* | Oleaceae | Lamiales | NC_008407 |
| 16 | *Lactuca sativa* | Asteraceae | Asterales | NC_007578 |
| 17 | *Liquidambar formosana* | Altingiaceae | Saxifragales | NC_023092 |
| 18 | *Lotus japonicus* | Fabaceae | Fabales | NC_002694 |
| 19 | *Lychnis chalcedonica* | Caryophyllaceae | Caryophyllales | NC_023359 |
| 20 | *Manihot esculenta* | Euphorbiaceae | Malpighiales | NC_010433 |
| 21 | *Medicago truncatula* | Fabaceae | Fabales | NC_003119 |
| 22 | *Morus indica* | Moraceae | Rosales | NC_008359 |
| 23 | *Nelumbo nucifera* | Nelumbonaceae | Proteales | NC_015610 |
| 24 | *Nicotiana tabacum* | Solanaceae | Solanales | NC_001879 |
| 25 | *Oenothera biennis* | Onagraceae | Myrtales | NC_010361 |
| 26 | *Panax ginseng* | Araliaceae | Apiales | NC_006290 |
| 27 | *Phaseolus vulgaris* | Araliaceae | Fabales | NC_009259 |
| 28 | *Populus trichocarpa* | Salicaceae | Malpighiales | NC_009143 |
| 29 | *Quercus rubra* | Fagaceae | Fagales | NC_020152 |
| 30 | *Solanum tuberosum* | Solanaceae | Solanales | NC_008096 |
| 31 | *Spinacia oleraceae* | Amaranthaceae | Caryophyllales | NC_002202 |
| 32 | *Vitis vinifera* | Vitaceae | Vitales | NC_007957 |
