# Supplementary material for: Analysis of the Complete Chloroplast Genome of a Medicinal Plant, Dianthus superbus var. longicalyncinus, from a Comparative Genomics Perspective
Source: PLoS One. 2015 Oct 29;10(10):e0141329. doi: 10.1371/journal.pone.0141329 (PMC4626046; doi:10.1371/journal.pone.0141329)
Supplement: S2 Table — (DOCX) [file pone.0141329.s006.docx]

**S2 Table**. **Prediction of RNA editing by the PREP-cp program.**

| Gene | Nucleotide position | Triplet position | Bases | Codon change | Amino acid change |
| --- | --- | --- | --- | --- | --- |
| *atpA* | 373 | 1 | C – U | CCU – UCU | P – S |
|  | 914 | 2 | C – U | UCA – UUA | S – L |
|  | 1270 | 1 | C – U | CCC – UCC | P – S |
| *accD* | 74 | 2 | C – U | ACG – AUG | T – M |
| *clpP* | 508 | 1 | C – U | CCG – UCG | P – S |
| *matK* | 637 | 1 | C – U | CAU – UAU | H – Y |
|  | 824 | 2 | C – U | GCU – GUU | A – V |
| *ndhA* | 341 | 2 | C – U | UCA – UUA | S – L |
|  | 566 | 2 | C – U | UCA – UUA | S – L |
|  | 1073 | 2 | C – U | UCC – UUC | S – F |
| *ndhB* | 149 | 2 | C – U | UCA – UUA | S – L |
|  | 467 | 2 | C – U | CCA – CUA | P – L |
|  | 542 | 2 | C – U | ACG – AUG | T – M |
|  | 586 | 1 | C – U | CAU – UAU | H – Y |
|  | 611 | 2 | C – U | UCA – UUA | S – L |
|  | 737 | 2 | C – U | CCA – CUA | P – L |
|  | 746 | 2 | C – U | UCU – UUU | S – F |
|  | 830 | 2 | C – U | UCA – UUA | S – L |
|  | 836 | 2 | C – U | UCA – UUA | S – L |
|  | 1481 | 2 | C – U | CCA – CUA | P – L |
| *ndhD* | 2 | 2 | C – U | ACG – AUG | T – M |
|  | 26 | 2 | C – U | ACA – AUA | T – I |
|  | 67 | 1 | C – U | CUU – UUU | L – F |
|  | 125 | 2 | C – U | UCA – UUA | S – L |
|  | 887 | 2 | C – U | CCC – CUC | P – L |
|  | 1298 | 2 | C – U | UCA – UUA | S – L |
|  | 1310 | 2 | C – U | UCA – UUA | S – L |
|  | 1360 | 1 | C – U | CUU – UUU | L – F |
|  | 1397 | 2 | C – U | ACU – AUU | T – I |
|  | 1460 | 2 | C – U | GCU – GUU | A – V |
| *ndhF* | 290 | 2 | C – U | UCA – UUA | S – L |
|  | 586 | 1 | C – U | CUU – UUU | L – F |
|  | 1777 | 1 | C – U | CCC – UCC | P – S |
| *ndhG* | 314 | 2 | C – U | ACA – AUA | T – I |
|  | 344 | 2 | C – U | ACU – AUU | T – I |
|  | 1787 | 2 | C – U | CCU – CUU | P – L |
| *petB* | 418 | 1 | C – U | CGG – UGG | R – W |
| *psaI* | 28 | 1 | C – U | CUU – UUU | L – F |
| *rpoB* | 473 | 2 | C – U | UCA – UUA | S – L |
|  | 551 | 2 | C – U | UCA – UUA | S – L |
| *rpoC1* | 638 | 2 | C – U | ACU – AUU | T – I |
| *rpoC2* | 1493 | 2 | C – U | ACG – AUG | T – M |
|  | 2267 | 2 | C – U | GCC – GUC | A – V |
| *rps2* | 248 | 2 | C – U | UCA – UUA | S – L |
| *rps14* | 80 | 2 | C – U | UCA – UUA | S – L |
