# Supplementary material for: Analysis of the Complete Chloroplast Genome of a Medicinal Plant, Dianthus superbus var. longicalyncinus, from a Comparative Genomics Perspective
Source: PLoS One. 2015 Oct 29;10(10):e0141329. doi: 10.1371/journal.pone.0141329 (PMC4626046; doi:10.1371/journal.pone.0141329)
Supplement: S3 Table — (DOCX) [file pone.0141329.s007.docx]

**S3 Table. The nucleotide sequences of *rpl36*-*rps8* and *rpl23* regions of *Dianthus* cp genome.**

>Dianthus_rpl36-rps8 regions (535 bp)

AAATTTTATGTTATATATGGTAATCCCTCTAATATCAAATATCTGAATTAGATCCAAAATTGCCTCTATTGTGAACAAAAAAAGACAAAGGACAAGTTCTCTAATATCTTTAATCTATTAGTTAATCTGTTAAGGAGGTTTTACCTGGAATGAAAGAACAAAAAACGATTCATTATAATTTGATTACTCAATCGCTCCCTAACGGTATGTTCTGGGTTCCCCTAGATTTTTTATAATGAAAATTGGATTGTATGTTTTATTTCCTAAAAGATACGCCGTAGTTCTATCCAGGCCCTACTGGGGGGGAAAATTGAAATAAGCCTTAGGTTATAGATATAATTTATAGACTTCGCACTAAGGATTCAAATGATTAAGTGGTTTTTCAACTCCAACACGCTTTCTCGCGAGAATACAATTCAAAATTTCAAATATCAAGAAACTTATTTTCTTCCAAGAACTAGATTCAGAATTTAAATTTAAAGTAAGGAAAAAATATGAAAATAAGGGCTTCTGTTCGTAAAATTTGTGAAAAATG

>Dianthus_rpl2-trnI-GAU regions (675 bp)

GTATTCTATGGTTACGATTCTACCATATATGTCTTTTTCATTCCGTCGAAAATCTATTTTACGGTATAGACGCTTATGACCTCCCCCCCTATGCCTTGCGGTAATGATGCCTCTGGCATTACGTCCTTTACCACAACGACGCTGTCCATAGATCAAATTATTTCGTGGATTGGATTTCACTTGATTGTCTACGGCTCCATTGCGTGTGCTCGGGGTAGAAGTTTTGTATAAATGTATCGCCATGCTATTAAGTATTTTTTTTTAAGTTATTTTCTTTCTAAGAGGTGGAATAGAATAACCCGATTGAAGCGTAATGATCATACGTCTGTAATGCATTGTATGTTCCATAATAGGTCCCATTCTTCTACCCTTTCGGAGGAGTCGATGACTATTACCTTGACACCAAAGAAGAGTTCGACCCAATACTTTATTTCCGTGCTAGTTGATTCGAATTCGACATTAGAAGTATATTGCTTTTTCCCCAATAACCGAATACTTTTGTCTGTAAATACTGCCTATTTGATTCCATCCATAAATCGAATTTCTTCCCTATGAGTTCGAGTCTCAATAAGAATGCTCGTTTTTTCTGTTCATATGTTATGTTATGATATGAATATACCATACCAATTCCTTATGTATGGTATATTCATATCATACCAATTCCTTATGTATG
